# Successful retrieval of a misdeployed stent using a drill dilator during endoscopic ultrasound-guided hepaticogastrostomy: a useful technique with a potential pitfall

**DOI:** 10.1055/a-2904-0208

**Published:** 2026-07-13

**Authors:** Kohei Okamoto, Norimitsu Uza, Masahiro Tsujimae, Arata Sakai, Takashi Kobayashi, Atsuhiro Masuda, Yuzo Kodama

**Affiliations:** 1 Division of GastroenterologyDepartment of Internal Medicine 592910 Kobe University Graduate School of MedicineKobeHyogo PrefectureJapan

Endoscopic ultrasound-guided biliary drainage (EUS-BD) is widely performed; however, unexpected complications occasionally occur. We report a case of EUS-guided hepaticogastrostomy (EUS-HGS) complicated by misdeployment of a plastic stent (PS) into the working channel of the endoscope, with successful retrieval using a drill dilator ( Video 1 ).

**Video 1** Successful retrieval of a misdeployed stent using a drill dilator: a useful technique with a potential pitfall.


**Fig. 1:**
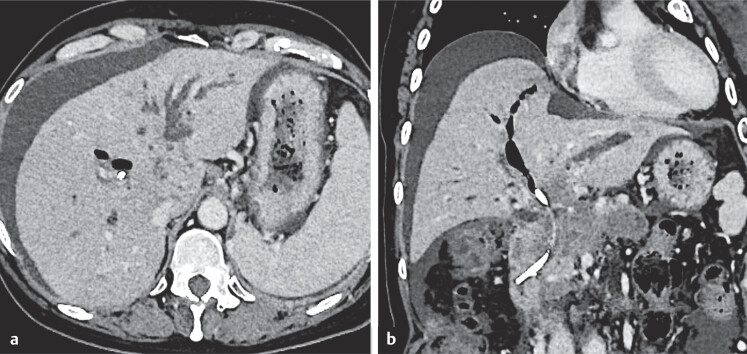
Computed tomography images before EUS-guided hepaticogastrostomy. ( **a** ) Drainage of the right lobe was performed transpapillary. ( **b** ) Drainage of the left lobe was not achieved because of severe hilar stricture caused by liver metastases.

**Fig. 2:**
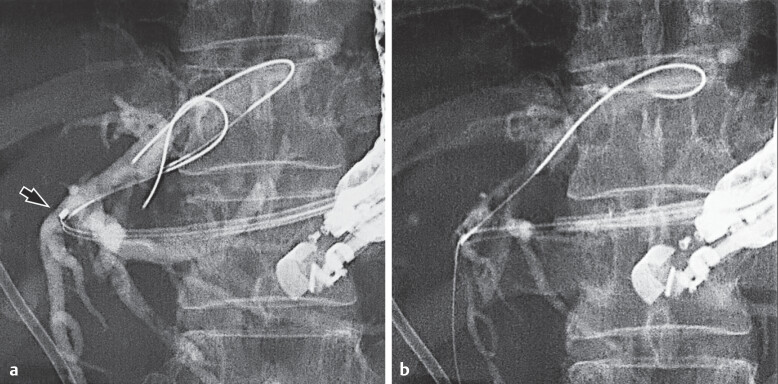
Fluoroscopic images. ( **a** ) A 7-Fr plastic stent could not be advanced sufficiently into the bile duct; the black arrowhead indicates the radiopaque marker at the tip of the inner sheath. ( **b** ) The plastic stent was misdeployed during the withdrawal of the delivery system; the radiopaque marker at the tip of the inner sheath was no longer visible.

**Fig. 3:**
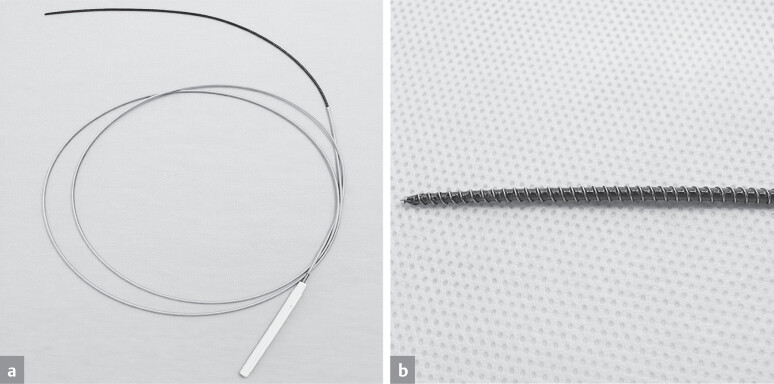
Tornus ES, a drill dilator. ( **a** ) An overall view of the device, showing the coil sheath and rotatable handle. ( **b** ) The tapered, drill-shaped distal tip enabling screw-in engagement with the plastic stent.

**Fig. 4:**
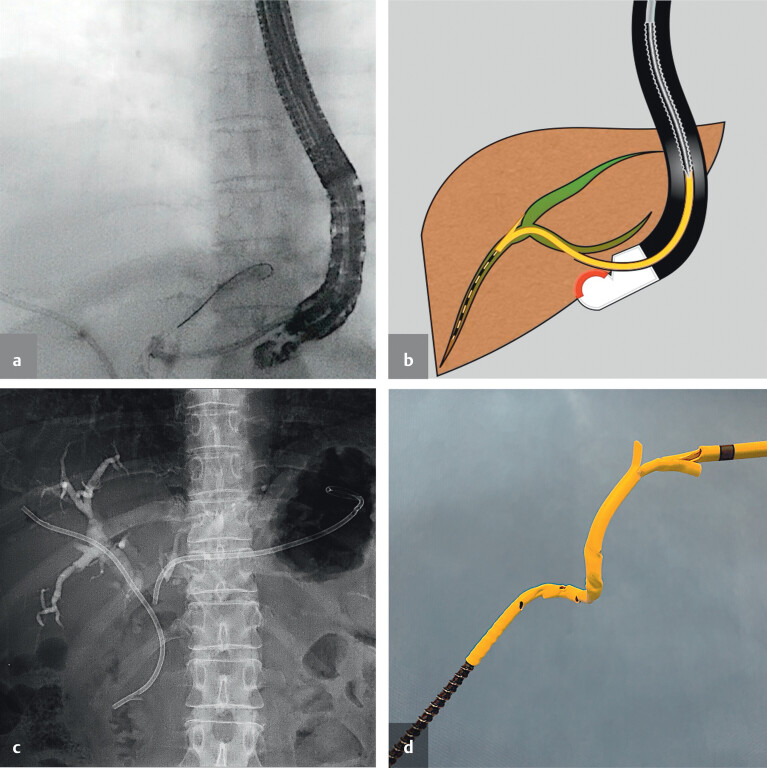
Tornus-assisted retrieval of the misdeployed stent and new stent placement. ( **a** ) The Tornus was not definitively confirmed fluoroscopically within the working channel. ( **b** ) Schematic illustration of the Tornus firmly screwed into the misdeployed plastic stent. ( **c** ) The successful placement of a new plastic stent. ( **d** ) Partial tearing of the retrieval stent where the Tornus had been screwed in.

A 52-year-old woman developed obstructive jaundice due to hilar metastases from pancreatic cancer. Transpapillary drainage of the left lobe failed, and we therefore performed EUS-HGS ( Fig. 1 ). We punctured B3 and placed two 0.025-in guidewires (GWs) using an uneven double-lumen cannula (Piolax, Tokyo, Japan). 1​ Because of ascites, we attempted to deploy a 7-Fr PS without tract dilation, but it could not be advanced sufficiently. During the withdrawal of the delivery system, the PS was misdeployed ( Fig. 2 ), presumably because its flap stuck in a side branch. 2​ Its proximal end was inadvertently released into the working channel; therefore, we inserted a drill dilator (Tornus ES, Olympus Co., Tokyo, Japan) over the GW ( Fig. 3 ). Although fluoroscopic imaging could not definitively confirm the Tornus within the working channel, it was successfully screwed into the stent ( Fig. 4a, b ), allowing the retrieval of the stent–dilator complex. Consequently, GW access was preserved and repuncture was avoided, thereby enabling the placement of a new PS ( Fig. 4c ). Notably, ex vivo examination revealed partial tearing of the PS where the Tornus had been screwed as shown in Fig. 4d . The patient’s jaundice rapidly improved, with no procedure-related adverse events.

The Tornus, primarily designed for tract dilation during EUS-BD, has been reported for the retrieval of migrated stents. 3​ ​4​ In this case, the Tornus was particularly effective because it could be screwed into the stent within the narrow working channel where conventional retrieval using grasping forceps or snares is difficult. However, excessive screwing may cause stent disruption, potentially precluding successful retrieval. This pitfall should be recognized during the Tornus-assisted retrieval of migrated stents; careful attention to tactile feedback and stent mobility is essential.

Endoscopy_UCTN_Code_TTT_1AS_2AD
